# Genome-wide identification of WRKY45-regulated genes that mediate benzothiadiazole-induced defense responses in rice

**DOI:** 10.1186/1471-2229-13-150

**Published:** 2013-10-04

**Authors:** Akira Nakayama, Setsuko Fukushima, Shingo Goto, Akane Matsushita, Masaki Shimono, Shoji Sugano, Chang-Jie Jiang, Aya Akagi, Muneo Yamazaki, Haruhiko Inoue, Hiroshi Takatsuji

**Affiliations:** 1 Disease Resistant Crops Research Unit, National Institute of Agrobiological Sciences, Ibaraki 305-8602, Japan; 2 Maebashi Institute of Technology, Maebashi 371-0816, Japan; 3 Present address: Du Pont Kabushiki Kaisha, 2-11-1, Nagata-cho, Chiyoda-ku, Tokyo 100-6111, Japan; 4 Present address: Department of Plant Pathology, Michigan State University, East Lansing, MI 48824, USA; 5 Present address: Bayer CropScience, Tokyo 100-8262, Japan; 6 Disease Resistant Crops Research Unit, National Institute of Agrobiological Sciences, 2-1-2 Kannondai, Tsukuba, Ibaraki 305–8602, Japan

**Keywords:** WRKY, Salicylic acid, Benzothiadiazole, *Magnaporthe oryzae*, OsNPR1

## Abstract

**Background:**

The rice transcription factor WRKY45 plays a crucial role in salicylic acid (SA)/benzothiadiazole (BTH)-induced disease resistance. Its knockdown severely reduces BTH-induced resistance to the fungal pathogen *Magnaporthe oryzae* and the bacterial pathogen *Xanthomonas oryzae* pv. *oryzae* (*Xoo*). Conversely, overexpression of WRKY45 induces extremely strong resistance to both of these pathogens. To elucidate the molecular basis of WRKY45-dependent disease resistance, we analyzed WRKY45-regulated gene expression using rice transformants and a transient gene expression system.

**Results:**

We conducted a microarray analysis using *WRKY45*-knockdown (*WRKY45*-kd) rice plants, and identified WRKY45-dependent genes among the BTH-responsive genes. The BTH-responsiveness of 260 genes was dependent on WRKY45. Among these, 220 genes (85%), many of which encoded PR proteins and proteins associated with secondary metabolism, were upregulated by BTH. Only a small portion of these genes overlapped with those regulated by OsNPR1/NH1, supporting the idea that the rice SA pathway branches into WRKY45- regulated and OsNPR1/NH1-regulated subpathways. Dexamethazone-induced expression of myc-tagged WRKY45 in rice immediately upregulated transcription of endogenous *WRKY45* and genes encoding the transcription factors WRKY62, OsNAC4, and HSF1, all of which have been reported to have defense-related functions. This was followed by upregulation of defense genes encoding PR proteins and secondary metabolic enzymes. Many of these genes were also induced after *M*. *oryzae* infection. Their temporal transcription patterns were consistent with those after dexamethazone-induced *WRKY45* expression. In a transient expression system consisting of particle bombardment of rice coleoptiles, WRKY45 acted as an effector to trans-activate reporter genes in which the luciferase coding sequence was fused to upstream and intragenic sequences of *WRKY62* and *OsNAC4*. Trans-activation of transcription occurred through a W-box-containing sequence upstream of *OsNAC4* and mutations in the W-boxes abolished the trans-activation.

**Conclusions:**

These data suggest a role of WRKY45 in BTH-induced disease resistance as a master regulator of the transcriptional cascade regulating defense responses in one of two branches in the rice SA pathway.

## Background

The salicylic acid (SA) defense signaling pathway plays a crucial role in mediating induced defense responses, including systemic acquired resistance. In Arabidopsis, the transcriptional coactivator NPR1 plays a key role in regulating the SA signaling pathway [1]. Rice has an NPR1 counterpart, OsNPR1/NH1 (denoted as OsNPR1 hereafter), which plays a major role in the resistance to blast and leaf blight diseases caused by the fungal pathogen *Magnaporthe oryzae* and the bacterial pathogen *Xanthomonas oryzae* pv. *oryzae* (*Xoo*), respectively [2,3]. Chemical inducers of defense known as 'plant activators’ can protect plants against various diseases by acting on the plant SA signaling pathway [4-8]. Plant activators confer protection against a wide range of pathogens, and their 'priming effect’ alleviates the costs of defense reactions on plant growth [9].

The WRKY transcription factors (TFs) form a super family that is involved in various regulatory processes in plants. They function via binding to a *cis*-element known as the “W-box” that is present in the promoters of target genes [10-12]. Several studies have highlighted the importance of WRKY TFs in transcriptional reprogramming of plant responses to different invading pathogens in various plant species [13]. The WRKY family has 109 members in rice [14]. Of these, several have been implicated in defense responses related to the SA pathway, mostly based on experimental evidence from overexpression studies [15-18]. However, the precise functions of most WRKY TFs remain unknown.

Previously, we identified rice WRKY45, which is transcriptionally inducible by the plant activator benzothiadiazole (BTH) [19]. WRKY45 belongs to group III of the WRKY family [20,21] and its closest Arabidopsis homolog is AtWRKY70 [22]. WRKY45 plays a crucial role in resistance to the important rice pathogens *M*. *oryzae* and *Xoo*, and is induced by various plant activators [19,23]. In our previous studies, overexpression of WRKY45 conferred extremely strong resistance to both *M*. *oryzae* and *Xoo*[19,23]. Microscopic analyses demonstrated that the blast resistance in *WRKY45*-overexpressing (*WRKY45*-ox) rice is based on a two-layered mechanism: pre-invasive defense, which prevents the invasion of fungal hyphae into rice cells, and post-invasive defense, which accompanies HR cell death, whereas probenazole-treated rice plants showed only the post-invasive defense [23]. We have shown that WRKY45 is regulated by ubiquitin-proteasome degradation, similar to Arabidopsis NPR1 [24]. We proposed that this regulation could play a role in suppressing unnecessary defense activation in the absence of pathogens [25]. Recently, we reported that WRKY45 also plays a role in blast resistance mediated by *Panicle blast 1*, a blast resistance gene with a coiled-coil nucleotide binding leucine-rich repeat structure, through a protein–protein interaction [26].

In Arabidopsis, NPR1 plays a major role in the SA pathway by regulating more than 99% of SA/BTH-regulated genes [27], while NPR1-independent pathway(s) also operate during early phases of pathogen infection [22,28,29]. However, experimental evidence suggests that the SA pathway in rice differs from that in Arabidopsis. Based on the results of epistasis analyses, we proposed that the SA-signaling pathway in rice branches into WRKY45-dependent and OsNPR1-dependent sub-pathways as a consequence of evolutionary divergence between rice and Arabidopsis [19,30]. Arabidopsis NPR1 equally regulates upregulation and downregulation of genes [27], but the molecular events that are regulated downstream of WRKY45 in rice are largely uncharacterized.

To elucidate the molecular basis of WRKY45-dependent disease resistance, we analyzed WRKY45-regulated gene expression using rice transformants in which *WRKY45* expression was knocked-down by RNAi. Then, we conducted further expression analyses of representative genes after chemical-induced *WRKY45* expression and *M*. *oryzae* infection. To validate the transcriptional cascade regulated by WRKY45, we also conducted trans-activation assays of TF genes.

## Results

### Identification of WRKY45-dependent BTH-responsive genes in rice

**Figure 1:**
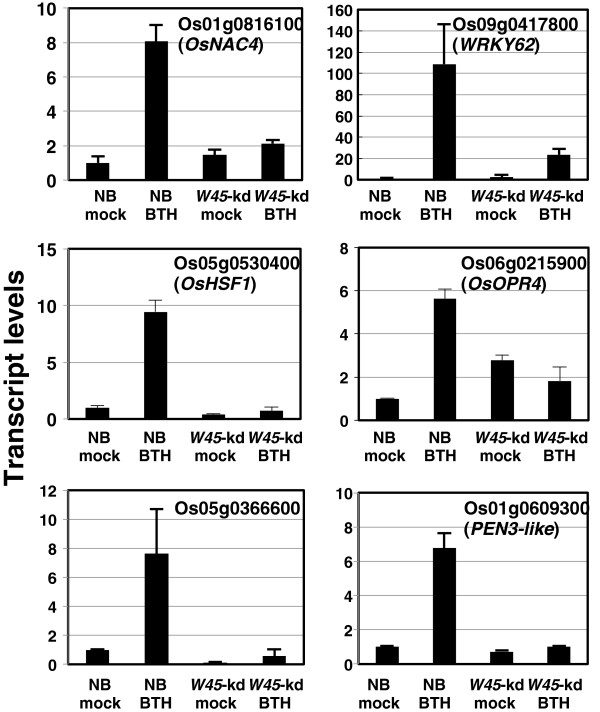
**qRT**-**PCR analysis to determine WRKY45-dependence of BTH-responsive genes.** The same RNAs samples (line #3) used for microarray analysis in Additional file 1 were analyzed by qRT-PCR to determine transcription patterns of some selected genes to validate BTH-responsiveness and WRKY45-dependence of expression determined by microarray analysis. Transcript levels of genes in mock- and BTH-treated NB and *WRKY45*(*W45*)-kd plants (relative to mock-treated NB) are shown with standard deviations (SD, *n* = 3). *Rubq1* was used as an internal control.

To identify WRKY45-dependent BTH-responsive genes that possibly play a role in BTH-induced disease resistance, we performed global gene expression analysis in Nipponbare (NB) and two lines (#3 and #15) of *WRKY45*-kd rice plants using a rice oligo DNA microarray covering 29,923 genes. In a previous study, we showed that rice resistance to blast and leaf-blight diseases induced by plant activators was partially compromised in these *WRKY45*-kd lines [23]. Comparison of gene expression profiles between BTH- and mock-treated NB with four biological replicate experiments, followed by analysis of variance (ANOVA), identified 1,510 BTH-responsive genes (*p* value <0.05, >2-fold change in expression). Two-way ANOVA of differential expression with respect to treatment (BTH or mock) and genotype (NB and *WRKY45*-kd #3) identified 881 genes as statistically significant WRKY45-dependent BTH-responsive genes [i.e., those with a q value < 0.2 after applying false discovery rate (FDR) multiple testing correction [31]. Among these 881 genes, 277 showed >2-fold changes between BTH-treated NB and line #3 *WRKY45*-kd plants. Of these, 260 genes were also statistically significant WRKY45-dependent BTH-responsive genes (q value < 0.2) in line #15 *WRKY45*-kd plants [31]. These 260 genes were defined as WRKY45-dependent BTH-responsive genes (Additional file 1: Table S1). Of the WRKY45-dependent BTH-responsive genes, 220 genes (85%) were upregulated in response to BTH and only 40 genes were downregulated, consistent with our previous results indicating that WRKY45 is a transcriptional activator [19]. We conducted quantitative RT-PCR (qRT-PCR) analyses to quantify transcript levels of several selected genes, some of which were further characterized as described below. The qRT-PCR results validated the results of the microarray analysis (Figure 1).

### Functional categories of WRKY45-dependent BTH-responsive genes

The genes identified as WRKY45-dependent BTH-responsive genes were functionally categorized (Additional file 2). Several genes encoding pathogenesis-related (PR) proteins, such as chitinase, glucanase, and peroxidases, and those encoding leucine-rich-repeat-containing putative resistance (R) proteins were among them. Recent studies of nonhost resistance in the powdery mildew fungus–Arabidopsis pathosystem have identified some components (PEN1, PEN2, and PEN3) involved in pre-invasive resistance mechanisms [32-34]. Interestingly, a gene encoding a PEN3-like ABC transporter (Os01g0609300, Additional file 3), which was previously implicated in the abiotic stress response [35], was WRKY45-dependently upregulated. The WRKY45-dependent genes also included many genes that are possibly involved in secondary metabolism, including those encoding cytochrome P450 (15 genes, Additional file 2). Cytochrome P450s have been implicated in the biosynthesis of antimicrobial phytoalexins and metabolism of xenobiotics [36], as well as plant hormones. UDP-glucuronosyl/UDP-glucosyltransferase (GT) family proteins of family 1 [37] have been implicated in glycosylation of secondary metabolites and hormones [38,39]. We found 11 genes encoding family 1 GTs among the WRKY45-dependent genes (Additional file 2). One of these, salicylic acid glucosyltransferase (OsSGT1, Os09g0518200), contributes to plant-activator-induced disease resistance [40]. Orthologs of another WRKY45-dependent family 1 *GT* gene (Os01g0176000) are pathogen-inducible in Arabidopsis, and their mutants showed decreased resistance to *P*. *syringae* pv. tomato-*AvrRpm1*[41]. The rice genome contains 59 genes encoding putative glutathione S-transferases (GST), and 22 of them were WRKY45-dependently upregulated (Additional file 2). In addition to their antioxidant activity, GSTs have a role in detoxifying toxins by conjugating glutathione to substrates. Genes encoding allene oxide synthase and 12-oxophytodienoic acid reductase (OPR), which are enzymes in the octadecanoid pathway leading to the production of oxygenated fatty-acids (oxylipins), were also WRKY45-dependently upregulated. The role of the oxylipin jasmonic acid (JA) in plant defense is well known; however, recent studies have revealed that oxylipins other than JA actively participate in plant defense mechanisms [42]. Interestingly, *OsOPR7*, which is responsible for JA synthesis [43], was not among the three WRKY45-dependently upregulated *OsOPRs* (Additional file 2). The substrates and products of these OsOPRs and the end products of their biosynthetic pathways remain unknown.

Several genes encoding TFs were WRKY45-dependently upregulated by BTH (Additional file 2). In rice, WRKY62 was reported to negatively regulate basal and Xa21-mediated resistance to bacterial blight disease [44,45]. OsNAC4 is a positive regulator of programmed cell death associated with the hypersensitive reaction (HR) [46,47]. *OsHSF1* is the causative gene for a lesion mimic mutant, *spontaneous lesion 7* (*spl7*), and its product negatively regulates plant cell death through decreasing cellular ROS levels [48,49].

### Temporal gene expression patterns of WRKY45-dependent genes after dexamethasone-induced *WRKY45* expression

**Figure 2:**
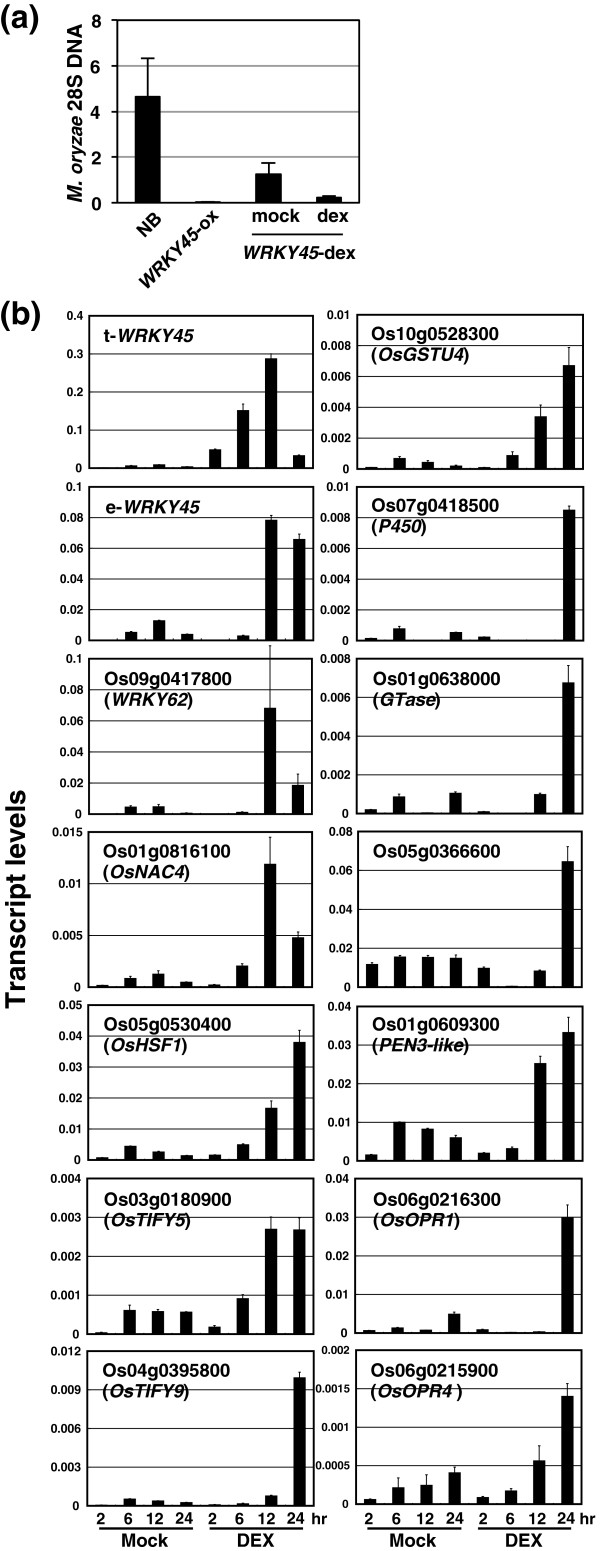
**Temporal expression patterns of WRKY45-regulated genes after DEX-induction of *WRKY45 *expression. (a)** Blast resistance in *WRKY45*-dex rice plants. Ten plants of T3 homozygous GVG-WRKY45-myc transformants (line #1) were treated with 60 μM DEX (in 0.2% [v/v] ethanol / 0.01% [v/v] Silwet L-77) or mock solution (solvent only), and then spray-inoculated with *M*. *oryzae* after 1 day. Plants overexpressing *WRKY45* under the control of the maize *ubiquitin* promoter [19] were tested for comparison. Amount of *M*. *oryzae* 28S ribosomal DNA (rDNA) relative to that of rice *ubiquitin 1* mRNA was determined 6 days after inoculation. Average values are shown with SD. **(b)** T3 homozygous GVG-WRKY45-myc transformants (line #1) were treated with DEX or mock solutions as described above. Fourth leaves were harvested from four plants per treatment at 2, 6, 12, and 24 h after treatment. Total RNA was extracted, and transcript levels of selected genes were analyzed by qRT-PCR. Average values from three experiments are shown with SD (*n* = 3). Mock treatment induced low levels of transcripts of the transgene and the candidate downstream gene; the reason for this is unclear.

To characterize the temporal patterns of WRKY45-dependent gene expression, we generated transgenic rice plants expressing C-terminally myc-tagged WRKY45 (WRKY45-myc) under the control of the dexamethasone (DEX)-inducible promoter (*WRKY45*-dex) [50]. To test whether the WRKY45-myc proteins were functional, we tested the *WRKY45*-dex transformant lines (T2 homozygotes) for blast resistance. The results showed that the *WRKY45*-dex plants were more resistant than untransformed Nipponbare to *M*. *oryzae*, even in the absence of DEX treatment, presumably due to leaky expression of *WRKY45*-*myc*. The resistance of *WRKY45*-dex plants was further enhanced by DEX treatment (Figure 2a). These results confirmed that the WRKY45-myc proteins retained their functions. *WRKY45*-dex plants (lines #1 and #4) grown on soil were sprayed with 60 μM DEX or a mock solution at the 4- to 5-leaf stage, and the fourth leaves were harvested 2, 6, 12, and 24 h after each treatment. qRT-PCR analysis revealed that transgene-derived *WRKY45* transcripts (t-*WRKY45*) began to increase within 6 h after DEX treatment (Figures 2b and Additional file 4: Figure S2). Interestingly, transcript levels of endogenous *WRKY45* increased soon after the DEX-induced increase of t-*WRKY45* (Figure 2b), suggesting autoregulation of WRKY45. However, we did not observe transactivation of the *WRKY45* gene by WRKY45 in a transient expression system (see below), suggesting that the autoregulation was indirect or required an additional factor(s).

Other WRKY45-dependent BTH-responsive genes showed various temporal expression patterns. The genes encoding two TFs, *WRKY62* (AK067834) and *OsNAC4* (AK073848), were induced relatively early after the induction of t-*WRKY45*, almost concomitantly with endogenous *WRKY45*, while genes encoding another two TFs, *OsHSF1* (AK100412) and *OsTIFY5* (AK073589), were induced later (Figure 2). We also analyzed the expression of other genes randomly selected from other functional groups. Some of these genes were upregulated later than endogenous *WRKY45*, with some [e.g., cytochrome P450 (AK072220) and OsOPR1 (AK103067)] activated later than others [e.g., OsGSTU4 (AK103453) and OsOPR4 (AK072596)] (Figure 2). A similar expression analysis using another independent transgenic line (Additional file 4) gave consistent results.

### Responses of WRKY45-regulated genes to *M*. *oryzae* infection

**Figure 3:**
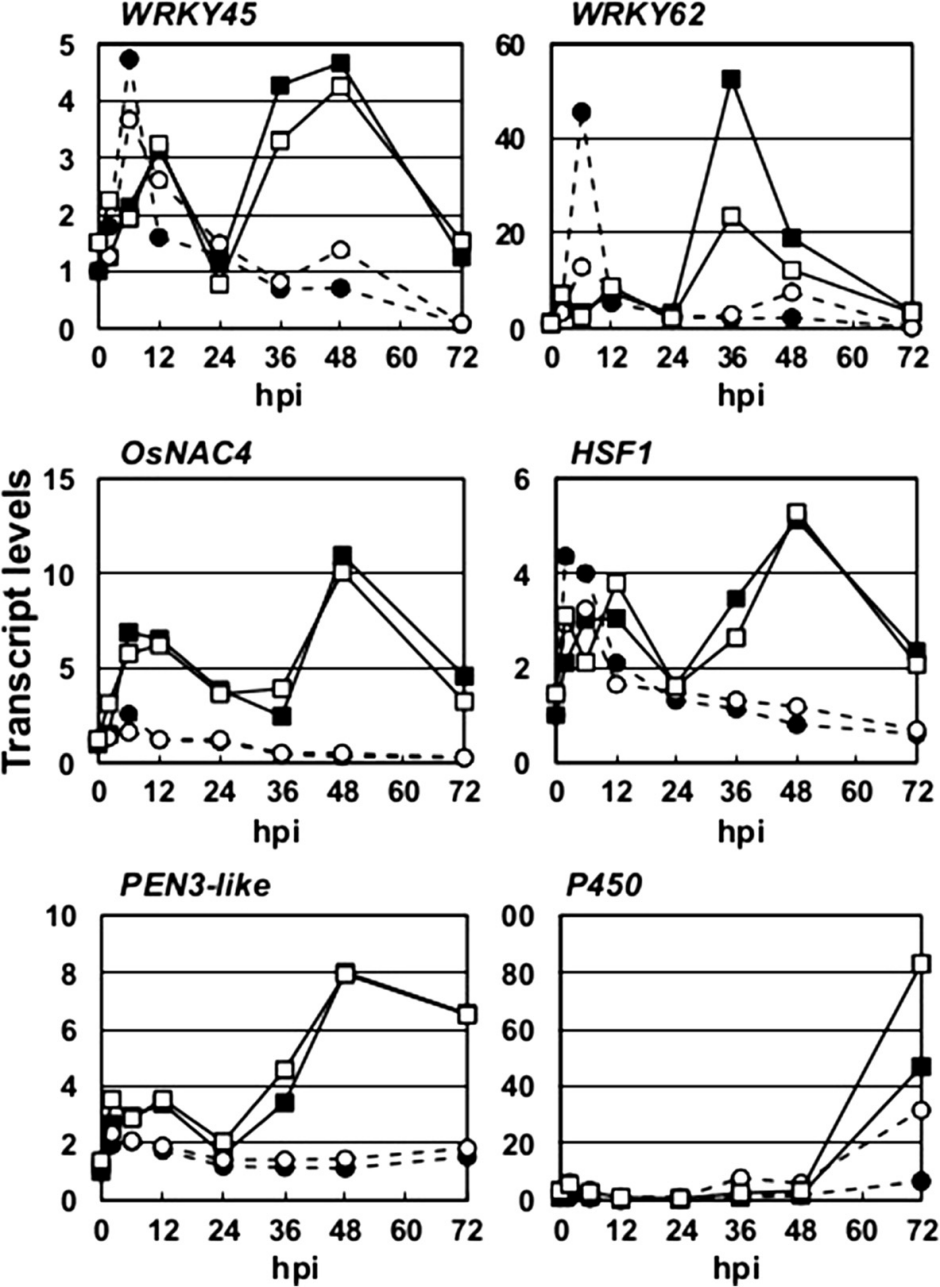
**Expression patterns of WRKY45-regulated genes in response to blast infection.** Leaves of NB rice plants were sprayed with *M*. *oryzae* conidia suspension or 0.02% Tween 20 (mock), and incubated at 25°C in the dark for indicated times. Changes in transcript levels of each gene over time were determined by qRT-PCR, relative to that of *Rubq1* as an internal standard. Results of two replicates each for *M*. *oryzae* inoculation (■, □) and mock treatment (●, ○) are shown.

We characterized the responses of some of the WRKY45-regulated genes to blast fungus infection in NB rice plants (Figure 3). Cut leaves of rice plants were inoculated with a compatible race of *M*. *oryzae* (race 007.0) or mock treated, and transcript levels of selected WRKY45-regulated genes were determined. The transcript level of *WRKY45* transiently and briefly increased in mock-treated leaves, presumably due to abiotic stresses such as high humidity, whereas *M*. *oryzae* inoculation delayed this early transcription of *WRKY45*. In addition, *M*. *oryzae* inoculation induced a second large peak of *WRKY45* transcription after 36 h. The transcription patterns of *WRKY62* and *OsHSF1* were similar to that of *WRKY45*. The transcription pattern of *OsNAC4* differed in that it was induced soon after *M*. *oryzae* inoculation and again after 36 h. *PEN3*-*like* (AK058981) and *P450* (AK072220) genes were induced later than were the genes encoding WRKY45 and other TFs. The timings of the transcription of these WRKY45-regulated genes relative to that of *WRKY45*, especially the timing of the second peak of transcription, were similar to those observed after DEX-induced expression of *WRKY45*. These results suggested that WRKY45 plays a major role in defense gene expression after *M*. *oryzae* infection.

### Transactivation of *OsNAC4* and *WRKY62* genes by WRKY45

**Figure 4:**
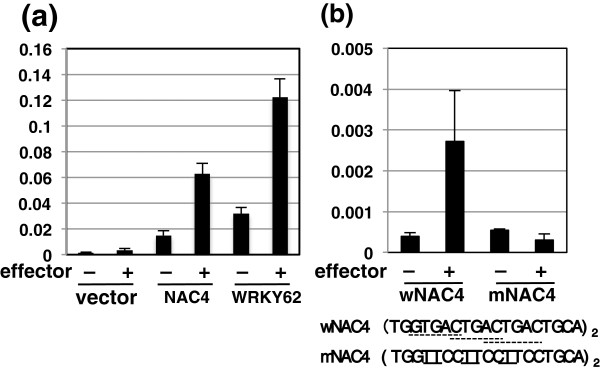
**Transactivation of *OsNAC4 *and *WRKY62 *genes by WRKY45. (a)** Transactivation of *WRKY62* and *OsNAC4* genes in transient trans-activation assays. Upstream and intragenic region of *WRKY62* (-1,070 – +1,838) or *OsNAC4* (-1,000 – +1,272) fused to the *hrLUC* gene in pGL4.7 vector (Promega) were used as reporter genes. Firefly *LUC* gene driven by the maize ubiquitin promoter was used as a reference gene. Reporter genes were co-delivered into rice coleoptiles with the effector genes (120 ng) with (+) or without (-) *WRKY45* cDNA, and the reference gene (firefly *LUC*). Luciferase activities were determined in three biological replicates. Average values are shown with SD. **(b)** Mutation analysis of W-boxes in *OsNAC4* upstream region in transient trans-activation assays. Duplicates of the *OsNAC4* upstream sequence (-504–-485) containing wild-type (wNAC4) or mutant (mNAC4) forms of W-box-like sequences fused to firefly *LUC* gene were used as reporter genes. Reporter genes were co-delivered with 500 ng of effector genes with (+) or without (-) WRKY45 cDNA described above, together with the *hrLUC* reference gene. Luciferase activities were determined in three replicates. Average values are shown with SD.

The DEX-inducible expression of WRKY45-myc proteins induced immediate upregulation of some WRKY45-dependent BTH-responsive genes encoding TFs besides WRKY45 (Figure 2). These results led us to examine the transactivation of their genes by WRKY45 using a transient expression assay in rice coleoptiles. Co-delivery of the *WRKY45* effector gene markedly activated reporter genes in which the *hrLUC* reporter gene was fused downstream of the upstream and intragenic sequences of the *OsNAC4* gene (Figure 4a). W-boxes (TTGACC/T) were present in the upstream region of *OsNAC4* at -498. Mutation of these W-boxes drastically reduced the transactivation activity, corroborating that these are the cis elements where WRKY45 interacts (Figure 4b). A W-box was also present in the upstream region of *WRKY62* at -454 (Figure 4a). However, this sequence did not mediate the activation of a reporter gene (data not shown). These results suggest a possibility that WRKY45 interacts with a non-W-box cis-element(s) or a W-box(s) downstream of the transcription start site in *WRKY62*. Alternatively, WRKY45 indirectly regulate *WRKY62*.

## Discussion

### WRKY45 regulates many BTH-responsive genes

To understand the functions of a particular TF, it is important to identify the genes that are directly or indirectly regulated by it. Constitutive overexpression of TFs can produce neomorphic phenotypes that do not necessarily reflect genuine functions of the TFs investigated [51], because overexpressed TFs occasionally bind to nonphysiological targets. To avoid this problem, we used *WRKY45*-kd rice in combination with BTH treatment to identify WRKY45-regulated genes. Microarray-based screening identified more than 200 genes whose BTH-responsiveness was WRKY45-dependent (Additional files 1 and 2). Consistent with WRKY45 being a transcriptional activator [19], ~85% of these genes were upregulated in response to BTH. Considering the fact that BTH-induced resistance to fungal and bacterial diseases was compromised in *WRKY45*-kd rice, these results suggest that the cumulative effects of the functions of WRKY45-regulated genes culminate in strong defense reactions.

### Role of WRKY45 in the SA pathway in rice

In Arabidopsis, expression of more than 99% of the BTH-responsive genes is dependent on NPR1, and several WRKY TFs are transcriptionally regulated downstream of NPR1 [27]. Our previous epistasis analyses demonstrated that in rice, WRKY45 and OsNPR1 are largely independent of each other in the SA-signaling pathway [19]. According to our transcript profiling [30], two-thirds of BTH-responsive genes in the OsNPR1-dependent sub-pathway are BTH-downregulated. These downregulated genes include several genes involved in photosynthesis and protein synthesis, suggesting that one of the major roles of OsNPR1 is to relocate energy and resources from house-keeping cellular activities to defense activities [30]. This contrasts with WRKY45-dependent BTH-responsive genes identified in this study, in that ~85% of them were upregulated by BTH. Notably, most of them were independent of OsNPR1 (Additional files 1 and 2), whereas their presumed counterpart genes are NPR1-dependent in Arabidopsis [27]. These results were consistent with our proposal that the SA-signaling pathway in rice branches into WRKY45-dependent and OsNPR1-dependent sub-pathways [19]. Similar to NPR1, WRKY45 is degraded by the proteasome, whereas we did not observe degradation of OsNPR1 [25]. These differences also seem to reflect the roles of WRKY45 and OsNPR1 in rice.

Arabidopsis AtWRKY70 is phylogenetically close to rice WRKY45 [22,52] and is regulated downstream of NPR1 [27]. The induction of the *AtWRKY70* gene by SA is partially independent of NPR1 during the early phase of induction [22,29], but it becomes NPR1 dependent later [27]. This implies an evolutionary relationship between AtWRKY70 and rice WRKY45.

### WRKY45 may regulate pre-invasive defense genes

Our microscopic analysis revealed an effective pre-invasive defense response in *WRKY45*-ox rice plants; that is, the infection of compatible *M*. *oryzae* was strongly blocked before the invasion of fungal hyphae into rice cells [23]. This is interesting in light of the repertoire of WRKY45-regulated genes. In dicots, the PR proteins are secreted extracellularly through a protein secretary pathway during SAR [53,54] and during nonhost resistance [33]. The *WRKY45*-dependent upregulation of *PR* genes suggests the involvement of PR proteins in the WRKY45-dependent pre-invasive defense. Studies of nonhost resistance in Arabidopsis against barley powdery mildew (*Blumeria graminis* f. sp. *hordei*) identified some components of pre-invasive defense mechanisms [32-34]. Bednarek *et al*. [55] proposed that PEN3, an ABC transporter located in the plasma membrane, plays a role in transporting antimicrobial secondary metabolites (glucosinolates) across the plasma membrane, leading to their extracellular accumulation. Particular cytochrome P450s, GSTs, and GTases have also been implicated in the synthesis of antimicrobial secondary metabolites [55]. It is interesting to note that some genes for ABC transporters similar to PEN3, as well as several cytochrome P450s, GSTs, and GTases, were among the WRKY45-dependently regulated genes identified in this study. Rice lacks the glucosinolate pathway; however, similar defense mechanisms involving yet-to-be-identified antimicrobial compounds could be involved in the WRKY45-dependent pre-invasive defense response. A pre-invasive defense response was not observed in rice plants treated with probenazole under our experimental conditions [23]. Because of this, we cannot conclude that the induction of pre-invasive defense is intrinsic to the function of WRKY45 under natural conditions. Further research is necessary to address this issue.

### Possible role of WRKY45 in regulation of plant cell death

Programmed cell death associated with the HR is an important part of various antimicrobial defense systems. Reactive oxygen species (ROS) play a role in HR-associated defense mechanisms both as signaling molecules and as biochemical triggers of programmed cell death. Iwai *et al*. [5] reported that HR lesions formed on rice leaves when blast fungus was inoculated onto rice plants pretreated with probenazole. In a previous study, microscopic analyses showed that the post-invasive defense accompanying HR cell death occurred WRKY45-dependently in probenazole-treated rice plants after *M*. *oryzae* infection [23]. In light of these observations, it is particularly interesting that *OsNAC4* was regulated as a direct target of WRKY45 in response to BTH. Overexpression of *OsNAC4* cDNA in cultured rice cells led to HR cell death accompanied by the loss of plasma membrane integrity and the fragmentation of nuclear DNA [46]. Thus, it seems possible that OsNAC4 regulates HR cell death in disease resistance induced by plant activators. Protein phosphorylation was required for nuclear localization of OsNAC4 and HR cell death [46]. This might account for the occurrence of HR cell death only after fungus inoculation in probenazole-treated rice. OsNAC4 regulates HR cell death through its downstream genes, *OsHSP90* and *IREN*, which are involved in the loss of plasma membrane integrity and nuclear DNA fragmentation, respectively [46,47]. Neither *OsHSP90* nor *IREN* was found to be WRKY45-dependent in the microarray analysis (Additional file 1), nor were they upregulated after DEX-induced expression of myc-tagged WRKY45 (data not shown). These results are consistent with the fact that post-translational regulation is required for OsNAC4 to induce HR cell death [46] and are also consistent with the lack of HR cell death in *WRKY45*-ox rice in the absence of pathogen inoculation.

OsHSF1 is a TF whose dominant negative mutant *spl7* shows lesion mimic spontaneous cell death (Yamanouchi *et al*., 2002), in which ROS levels are increased (Kojo *et al*., 2006). Thus, the normal function of OsHSF1 is presumably to suppress plant cell death by protecting plant cells from oxidative cellular damage caused by ROS. Necrotic plant cell death in an extensive area of the plant body can be damaging, and is also beneficial for hemibiotrophic pathogens such as *M*. *oryzae*. Therefore, preventing necrotic plant cell death could be an important part of defense responses.

## Conclusions

**Figure 5:**
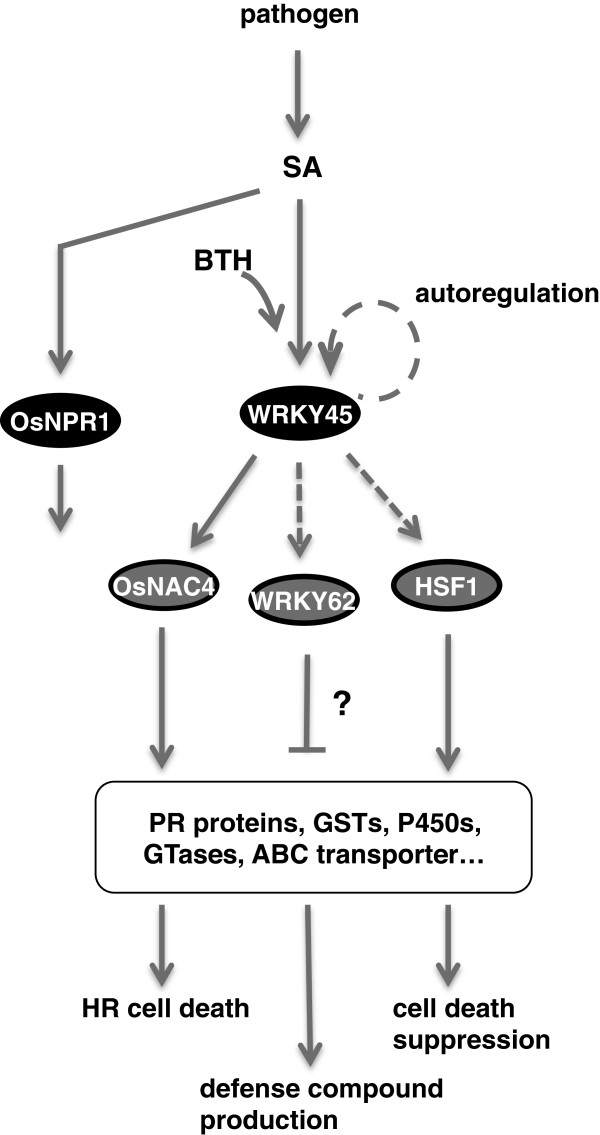
**Proposed model for a transcriptional cascade in rice SA pathway.** The SA pathway branches into WRKY45- and OsNPR1-dependent pathways. Transcription factors WRKY62, OsNAC4, and HSF1 mediate the SA signal downstream of WRKY45 to regulate defense genes. The gene encoding OsNAC4 is regulated directly by WRKY45, while the factors regulating genes encoding WRKY62 and HSF1 are unclear.

The repertoire of WRKY45-dependent genes among BTH-responsive genes is consistent with the idea that the rice SA pathway branches into two subpathways (Figure 5). The sequential expression of genes encoding transcription factors and defense genes, as well as the transactivation of TF genes by WRKY45, suggests a transcriptional cascade in which WRKY45 regulates the genes encoding downstream TFs, which in turn regulate different sets of defense genes (Figure 5). Overexpression of WRKY45 induced extremely strong resistance to both the fungal pathogen *M*. *oryzae* and the bacterial pathogen *Xoo*. The mighty potential of this TF is probably because it governs the SA-pathway-mediated defense responses as a master TF of this transcriptional cascade. The identification of WRKY45-regulated genes has provided some insights into the defense reactions regulated by WRKY45, together with the phenotypes of *WRKY45*-kd and -ox transformants. These analyses have also raised some questions, such as why *WRKY62*, which encodes a negative regulator of *Xoo* resistance [44], is regulated by WRKY45. Functional characterization of each downstream TF will help to dissect further the defense mechanisms regulated by WRKY45 and provide important information for its practical application.

## Methods

### Plant materials and chemicals

Rice (*Oryza sativa* ssp. *japonica* cv. Nipponbare) plants were grown in a growth chamber and treated with BTH as described previously [19]. For DEX treatments, 60 μM DEX solution (in 0.2% [v/v] ethanol / 0.01% [v/v] Silwet L-77 (OSI Specialties Inc., Danbury, CT, USA) was sprayed onto leaves of rice plants at the 4- to 5-leaf stage. For mock treatments, the solvent alone was sprayed onto plants.

### Plasmid construction and plant transformation

The plasmid to drive DEX-induced expression of myc-tagged WRKY45 proteins in rice cells was constructed as follows: the CDS of *WRKY45* was amplified by PCR with the primers *Xho*I-WRKY45FW (5’-CTCGAGATGACGTCATCGATGTC-3’) and *Bam*HI-WRKY45RV (5’-GGATCCAAAGCTCAAACCCATAATG-3’). The amplified fragment was then inserted into the pGEM-T Easy Vector (Promega, http://www.promega.com/) to generate pGEM-WRKY45, which contained the *WRKY45* CDS and a *BamH*I site between *Xho*I and *Sac*I sites. A DNA fragment encoding three tandemly repeated *myc* sequences (3 × *myc*, 5’-ATGGAGCAAAAGCTTATCAGTGAGGAAGACTTGAACGAGCAGAAGCTGATTTCCGAAGAGGATCTCAACGAGCAAAAGCTCATCTCGGAGGAAGACCTGCTC-3’) was inserted between the *BamH*I and *Sac*I sites in pGEM-WRKY45. A DNA linker containing an *Xba*I site was inserted between the *Sac*I and *Nsi*I sites in pGEM-WRKY45. Finally, a fragment encoding *WRKY45* CDS and 3 × *myc* sequence was excised with *Xho*I and *Xba*I, and inserted between *Xho*I and *Spe*I sites in a DEX-inducible gene expression vector, pTA7002 [50]. Rice plants were transformed via an *Agrobacterium* (strain EHA105)-mediated method [56] to generate transgenic GVG-WRKY45-myc plants.

### Microarray analysis

NB and *WRKY45*-knockdown (*WRKY45*-kd) rice plants grown in a growth chamber were treated with 0.5 mM BTH (in 0.5% [v/v] acetone / 0.05% [v/v] Tween 20) or a mock solution. Then, the fourth leaves were harvested from four plants from each treatment/genotype and pooled on the basis of different treatments and genotypes. Total RNA was isolated from each pool, labeled with Cy3, and hybridized to an Agilent Rice Oligo Microarray (44 k, custom-made, http://www.home.agilent.com). We analyzed four biological replicate sample sets for each treatment–genotype combination. Microarray experiments and data analyses were carried out as described previously [19,30]. Briefly, BTH-responsive genes were identified in NB rice, based on both significance [ANOVA *p*-value <0.05 after applying FDR multiple testing correction, according to the method of Benjamini and Hochberg [31]] and -fold change (2-fold). These genes were filtered through a two-way ANOVA, considering both genotype and treatment effects. WRKY45-dependent BTH-responsive genes were defined as those that showed decreases in BTH-responsive expression (to <1/2 of their corresponding expressions in NB) in two lines of *WRKY45*-kd rice (#3 and #15) with the q value < 0.2 after applying FDR multiple testing corrections [31].

### *M*. *oryzae* inoculation

Rice plants were grown for 20 days in a greenhouse. At the 4-leaf stage, the youngest leaves were cut and placed on wet paper in plastic cases. After preincubation at 25°C for 6 h, leaves were spray-inoculated with aqueous suspensions (5.0 × 10^5^ spores/ml in 0.02% Tween 20) of compatible *M*. *oryzae* conidia (race 007.0), and then kept in the dark for 3 days.

### qRT-PCR analysis

Total RNA was isolated from rice plants using Trizol reagent (Invitrogen, http://www.lifetechnologies.com) and purified with an RNeasy mini kit (Qiagen, http://www.qiagen.com). Reverse transcription was carried out using SuperScript II RNase H (Invitrogen) and oligo(dT)23 primers (Sigma-Aldrich, http://www.sigmaaldrich.com/). qRT-PCR was carried out on a Thermal Cycler Dice TP800 system (TaKaRa, http://www.takara-bio.co.jp/) using SYBR premix ExTaq mixture (TaKaRa) with cycles of 95°C for 5 s, 55°C for 10 s and 72°C for 20 s. *Rice ubiquitin 1* (*Rubq1*; AK121590) was used as an internal standard. The primers used are listed in Additional file 5.

### Transient expression assay

For transactivation assays, inner leaf sheaths of rice were cut into pieces and placed side-by-side on agar plates containing 0.4 M mannitol. The plasmids with or without *WRKY45* cDNA driven by the cauliflower mosaic virus *35S* promoter in the pUCAP vector were used as effector genes. The effector plasmids were introduced into the leaf sheaths together with reporter (3 μg) and reference (500 ng) genes containing *luciferase* (*LUC*) genes as specified using a PDS-1000/He Biolistic Particle Delivery system (Bio-rad, http://www.bio-rad.com). After incubation at 28°C for 6 h, samples were collected and ground in liquid nitrogen. Luciferase activities were assayed with the DualGlo Luciferase Reporter Assay system (Promega) and the ratio of the activity of the reporter gene product relative to that of the reference gene product was calculated.

### Availability of supporting data

The data set of microarray data supporting the results of this article are available in the Gene Expression Omnibus repository, GSE23733. http://www.ncbi.nlm.nih.gov/geo/query/acc.cgi?acc=GSE23733.

## Competing interests

The authors declare that they have no competing interests.

## Authors’ contributions

AN designed experiment, performed microarray and gene expression analysis and wrote manuscript. SG analyzed gene expression and tested blast resistance of plants. SF and AM performed transactivation experiments. MS and SS generated transformants. AA, MY, CJ and HI helped gene expression analysis. HT designed the study, interpreted the data, and wrote the manuscript. All authors read and approved the final manuscript.

## Supplementary Material

Additional file 1 BTH-inducible and WRKY45-dependent genes. Click here for file

Additional file 2 **Partial list of WRKY45**-**dependent BTH**-**responsive genes.** Some WRKY45-regulated BTH-responsive genes categorized according to function are shown with -fold changes in transcript level during BTH response (BTH/mock) in Nipponbare (NB), and WRKY45-dependence (BTH-treated NB/*WRKY45*-kd). Data are from *WRKY45*-kd rice line #3. OsNPR1-dependence of the BTH response of each gene [30] is also shown. Data set for all WRKY45-regulated BTH-responsive genes (277 genes) identified from *WRKY45*-kd line #3 plants is shown in Additional file 1: Table S1 online, as well as results obtained from *WRKY45*-kd line #15. Click here for file

Additional file 3 Sequence alignment of Arabidopsis PEN3 and rice PEN3-like proteins. Click here for file

Additional file 4 **Expression of WRKY45**-**regulated genes after DEX**-**induced*WRKY45*expression.** Expression of selected WRKY45-regulated genes in DEX-treated plants of T2 GVG-WRKY45-myc transformant line #4, a transformant line independent of that used in Figure 1, was analyzed by qRT-PCR as in Figure 1. Results were consistent with those in Figure 1. Click here for file

Additional file 5 Sequences of primers used in this study. Click here for file
