# Supplementary material for: Genome-wide identification of WRKY45-regulated genes that mediate benzothiadiazole-induced defense responses in rice
Source: BMC Plant Biol. 2013 Oct 4;13:150. doi: 10.1186/1471-2229-13-150 (PMC3850545; doi:10.1186/1471-2229-13-150)
Supplement: Additional file 3 — Sequence alignment of Arabidopsis PEN3 and rice PEN3-like proteins. [file 1471-2229-13-150-S3.pdf]

```
PEN3             1:  MDYNPNLPPL GGGGVSMRRS ISRSVSRASR NIEDIFSSGS RRTQSVNDDE EALKWAAIEK LPTYSRLRTT LMNAVVEDDV YGNQLMSKEV
                                                                                             **
Os01g0609300     1:  ---------- ---------- ---------- ---------- ---------- ---------- ---------- --NA------ ----------

                91:  DVTKLDGEDR QKFIDMVFKV AEQDNERILT KLRNRIDRVG IKLPTVEVRY EHLTIKADCY TGNRSLPTLL NVVRNMGESA LGMIGIQFAK
                                                             * * **                   **
                 3:  ---------- ---------- ---------- --------LG I-LP------ ---------- --NR------ ---------- ----------

               181:  KAQLTILKDI SGVIKPGRMT LLLGPPSSGK TTLLLALAGK LDKSLQVSGD ITYNGYQLDE FVPRKTSAYI SQNDLHVGIM TVKETLDFSA
                     *     * *  ** *** *** ****** *** *********  * * *  **   ****    * ***  * *** ** *** * * ** *** ***
                10:  KQTMPVLHDV SGIIKPRRMT LLLGPPGSGK TTLLLALAGR LGKDLKASGK VTYNGHGMEE FVPERTAAYI SQHDLHIGEM TVRETLAFSA

               271:  RCQGVGTRYD LLNELARREK DAGIFPEADV DLFMKASAAQ GVKNSLVTDY TLKILGLDIC KDTIVGDDMM RGISGGQKKR VTTGEMIVGP
                     ****** * *  * ** ****  * * * **  * **** *   *      ***  ****** **  ** *** *  ******* ** ****** ***
               100:  RCQGVGSRFD MLTELSRREK AANIKPDADI DAFMKAAAMG GQEANVNTDY ILKILGLEIC ADTMVGDEML RGISGGQRKR VTTGEMLVGP

               361:  TKTLFMDEIS TGLDSSTTFQ IVKCLQQIVH LNEATVLMSL LQPAPETFDL FDDIILVSEG QIVYQGPRDN ILEFFESFGF KCPERKGTAD
                        ******* ********** **  * * **     *   ** *******  * ****** * * ********    ****** ** *** *** **
               190:  ARALFMDEIS TGLDSSTTFQ IVNSLRQTVH ILGGTAVISL LQPAPETYNL FDDIILLSDG QIVYQGPRED VLEFFESMGF KCPDRKGVAD

               451:  FLQEVTSKKD QEQYWVNPNR PYHYIPVSEF ASRYKSFHVG TKMSNELAVP FDKSRGHKAA LVFDKYSVSK RELLKSCWDK EWLLMQRNAF
                     ********** * ***      **    * **  *   *** *     ****** ****  * ** *    *      ****    *  * *** ** *
               280:  FLQEVTSKKD QRQYWARHDK PYRFVTVKEF VSAFQSFHTG RAIANELAVP FDKSKSHPAA LATTRYGAPG KELLKANIDR EILLMKRNSF

               541:  FYVFKTVQIV IIAAITSTLF LRTEMNTRNE GDANLYIGAL LFGMIINMFN GFAEMAMMVS RLPVFYKQRD LLFYPSWTFS LPTFLLGIPS
                      * * * *      *  ***  ** *            * ***  **    *** ** * *  *   **** **** ***** *      *   * **
               370:  VYMFRTFQLM VVSLIAMTLF FRTKMKRDSV TSGGIYMGAL FFGVLMIMFN GFSELALTVF KLPVFFKQRD LLFYPAWSYT IPSWILKIPI

               631:  SILESTAWMV VTYYSIGFAP DASRFFKQFL LVFLIQQMAA SLFRLIASVC RTMMIANTGG ALTLLLVFLL GGFLLPKGKI PDWWGWAYWV
                        *        *** ***      **** * *   * ***  **** *     * *  **        **    * *** *          ** * **
               460:  TFIEVGGYVF LTYYVIGFDS NVGSFFKQYL LMLAINQMAG SLFRFIGGAA RNMIVANVFA SFMLLIFMVL GGFILAREQV KKWWIWGYWI

               721:  SPLTYAFNGL VVNEMFAPRW MNKMASSNST IKLGTMVLNT WDVYHQKNWY WISVGALLCF TALFNILFTL ALTYLNPLGK KAGLLPEEE-
                     **  ** *    ***     *      ** *    **  **     *     ** **  **   * * *** **** ***** * *        ***
               550:  SPMMYAQNAI SVNELMGHSW NKIVNSSASN ETLGVQVLKS RGVFPEARWY WIGFGAMIGF TILFNALFTL ALTYLRPYGN SRQSVSEEEL

               810:  -----NEDAD QGKDPMRRSL STADGNRRGE VAMG-RMSRD SAAEASGGAG NKKGMVLPFT PLAMSFDDVK YFVDMPGEMR DQGVTETRLQ
                          *        *    *  **    **      **     * *             ******* **  *** *  * **** **   ***   **
               640:  KEKRANLNGE IVGDVHLSSG ST----RR-- -PMGNGTEND STIVDDDTEV TQRGMVLPFT PLSLSFDNVR YSVDMPQEMK AQGVADDRLE

               894:  LLKGVTGAFR PGVLTALMGV SGAGKTTLMD VLAGRKTGGY IEGDVRISGF PKVQETFARI SGYCEQTDIH SPQVTVRESL IFSAFLRLPK
                     ***** * ** ********** ********** ********** ***   ***  ** ******  ****** *** ****** ***  *** ****
               723:  LLKGVSGSFR PGVLTALMGV SGAGKTTLMD VLAGRKTGGY IEGSINISGY PKKQETFARV SGYCEQNDIH SPQVTVYESL LFSAWLRLPE

               984:  EVGKDEKMMF VDQVMELVEL DSLRDSIVGL PGVTGLSTEQ RKRLTIAVEL VANPSIIFMD EPTSGLDARA AAIVMRAVRN TVDTGRTVVC
                      *      **    *******  ****  *** *** ****** ********** ********** ********** ****** *** ** *******
               813:  DVDSNTRKMF IEEVMELVEL KSLRDALVGL PGVNGLSTEQ RKRLTIAVEL VANPSIIFMD EPTSGLDARA AAIVMRTVRN TVNTGRTVVC

              1074:  TIHQPSIDIF EAFDELMLMK RGGQVIYAGP LGQNSHKVVE YFESFPGVSK IPEKYNPATW MLEASSLAAE LKLSVDFAEL YNQSALHQRN
                     ********** ****** *** ***  ***** **  *      **** ***** *   ****** ***      *   * ***    *  * * ***
               903:  TIHQPSIDIF EAFDELFLMK RGGEEIYAGP LGHHSSELIK YFESIPGVSK IKDGYNPATW MLEVTTIGQE QALGVDFSDI YKKSELYQRN

              1164:  KALVKELSVP PAGASDLYFA TQFSQNTWGQ FKSCLWKQWW TYWRSPDYNL VRFIFTLATS LLIGTVFWQI GGNRSNAGDL TMVIGALYAA
                     *** * ** *     *****  ** **    *    *****    *** * **  *** **     ** ** **   **      **     *  ***
               993:  KALIKDLSQP APDSSDLYFP TQYSQSSLTQ CMACLWKQNL SYWRNPPYNA VRFFFTTVIA LLFGTIFWDL GGKVTKSQDL FNAMGSMYAA

              1254:  IIFVGINNCS TVQPMVAVER TVFYRERAAG MYSAMPYAIS QVTCELPYVL IQTVYYSLIV YAMVGFEWKA EKFFWFVFVS YFSFLYWTYY
                       * *  **   *** ***** ********** **** ***   **  * ** *  *   *  ** *** **** *  ****  *    *  ** * *
              1083:  VLFIGVMNCT SVQPVVAVER TVFYRERAAG MYSAFPYAFG QVVIEIPYTL VQATVYGIIV YAMIGFEWTA AKFFWYLFFM VFTLLYFTFY

              1344:  GMMTVSLTPN QQVASIFASA FYGIFNLFSG FFIPRPKIPK WWIWYYWICP VAWTVYGLIV SQYGDVETRI QVLGGAPDLT VKQYIEDHYG
                     *** * ****    ***  ** ** * ***** * ****  *  ** ** * ** **** *** * ** ** **         * *    **   *   *
              1173:  GMMAVGLTPN YHIASIVSSA FYAIWNLFSG FVIPRPRVPI WWRWYCWACP VAWTLYGLVV SQFGDIET-- PMEDGTP--- VKVFVENYFG

              1434:  FQSDFMGPVA AVLIAFTVFF AFIFAFCIRT LNFQTR-
                     *     * **  *  **   * *  * * *    *** *
              1258:  FKHSWLGWVA TVVAAFAFLF ASLFGFAIMK FNFQKR*
```

Additional file 3. Sequnce alignment of *Arabidopsis* PEN3 and a rice PEN3-like protein
