# Supplementary material for: Genome-wide identification of WRKY45-regulated genes that mediate benzothiadiazole-induced defense responses in rice
Source: BMC Plant Biol. 2013 Oct 4;13:150. doi: 10.1186/1471-2229-13-150 (PMC3850545; doi:10.1186/1471-2229-13-150)
Supplement: Additional file 5 — Sequences of primers used in this study. [file 1471-2229-13-150-S5.doc]

Additional file 5. Primers used in this study

|  |  | Left primer | Right primer |
| --- | --- | --- | --- |
| qRT-PCR | t-*WRKY45* | CGGCATGGAGTTCTTCAAGAACGA | CGTTGTCGAAACCGATGATACGGA |
|  | e-*WRKY45* | CCACGCGTGTGTACAGAAAT | GACCCCCAGCTCATAATCAA |
|  | Os09g0417800 | TCCCACACATTCCTGTTCAT | ACTTGCAAGCATTTTGGGAT |
|  | Os01g0816100 | TGGATGGAGCAAGAAAAAGG | CCACCACATTTGCAGAATCA |
|  | Os05g0530400 | ATTTGCGCGGATTCATAGTT | ATGCACAGTAGTCAGCGGAT |
|  | Os03g0180900 | CGTGTCTGTGGAAAGTGTGG | GCTACTAATTCCCCCGGAAG |
|  | Os04g0395800 | CTGTTGATTTGGTCCCCTTG | ATTATTAACTGGCCCCTGCC |
|  | Os10g0528300 | TCCCATTGTTTGGGAGAAAA | GAATATTCCTTGCATTATTCAGACA |
|  | Os07g0418500 | TTCTATAAGAGTTTGCCACTTCG | CAAATACATTGTCTGAATAACATCAA |
|  | Os01g0638000 | AAGGTGATTGACGGCTTGAC | GGCGCCATAAATATAGTTACTCCA |
|  | Os06g0216300 | TCACCTAAATAATCCAATTTTAGCC | AAAATGTATTCATGCTTGTCCGT |
|  | Os06g0215900 | ACGAGGGCGCTGCTACTTAT | CGGATGGAAGTGAGGGTCA |
|  | Os05g0366600 | GCTTATCGGGAGTGCATGAT | TGAAAGAAATGTTCCACAAACG |
|  | Os01g0609300 | TTCATTGAGTTGCAGACGCT | GGGGAAAGGCTGGTATGATT |
|  | *Rubq1* | GGAGCTGCTGCTGTTCTAGG | TTCAGACACCATCAAACCAGA |
| plasmid construction | *Xho*I-WRKY45- *Bam*HI | CTCGAGATGACGTCATCGATGTC | GGATCCAAAGCTCAAACCCATAATG |
