# Supplementary material for: Genome-wide identification of WRKY45-regulated genes that mediate benzothiadiazole-induced defense responses in rice
Source: BMC Plant Biol. 2013 Oct 4;13:150. doi: 10.1186/1471-2229-13-150 (PMC3850545; doi:10.1186/1471-2229-13-150)
Supplement: Additional file 4 — Expression of WRKY45-regulated genes after DEX-inducedWRKY45expression. Expression of selected WRKY45-regulated genes in DEX-treated plants of T2 GVG-WRKY45-myc transformant line #4, a transformant line independent of that used in Figure 1, was analyzed by qRT-PCR as in Figure 1. Results were consistent with those in Figure 1. [file 1471-2229-13-150-S4.pdf]

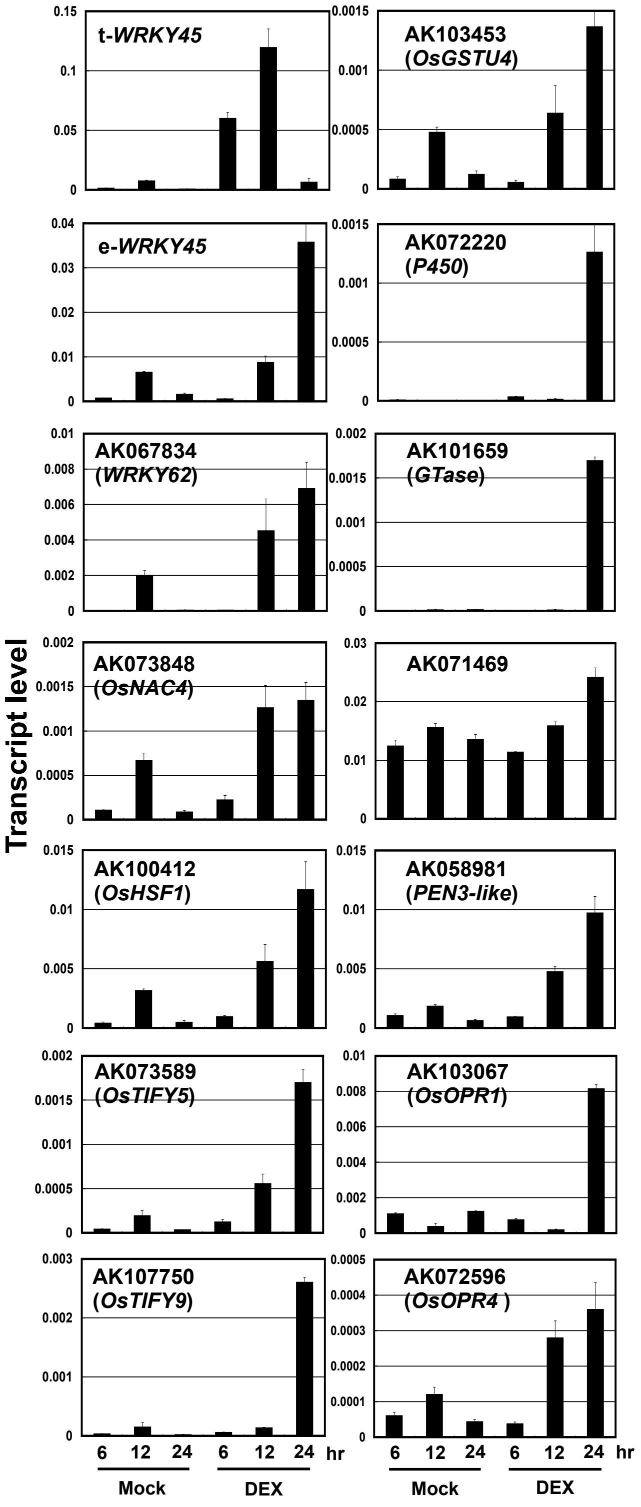

Additional file 4. Upregulation of WRKY45-regulated genes after DEX-induced *WRKY45* expression. Expression of selected WRKY45-regulated genes in DEX-treated plants of T2 GVG-WRKY45-myc transformant line #4, a transformant line independent of that used in Figure 1, was analyzed by qRT-PCR as in Figure 1. Results were consistent with those in Figure 1.
